# Maize Inoculation with Microbial Consortia: Contrasting Effects on Rhizosphere Activities, Nutrient Acquisition and Early Growth in Different Soils

**DOI:** 10.3390/microorganisms7090329

**Published:** 2019-09-07

**Authors:** Klára Bradáčová, Maximilian Sittinger, Katharina Tietz, Benjamin Neuhäuser, Ellen Kandeler, Nils Berger, Uwe Ludewig, Günter Neumann

**Affiliations:** 1 Institute of Crop Science (340h), Universität Hohenheim, Fruwirthstraße 20, 70593 Stuttgart, Germany (K.T.) (B.N.) (U.L.) (G.N.); 2 Julius Kühn-Institut, Institute for Biological Control, Heinrichstraße 243, 64287 Darmstadt, Germany; 3 Institute of Soil Science and Land Evaluation, Soil Biology Department, Universität Hohenheim, Emil-Wolff-Straße 27, 70593 Stuttgart, Germany; 4 EuroChem Agro GmbH, 8165 Mannheim, Germany

**Keywords:** plant–microbial interactions, plant growth-promoting microorganisms, P solubilization, microbial consortia, ammonium, auxin-responsive genes

## Abstract

The benefit of plant growth-promoting microorganisms (PGPMs) as plant inoculants is influenced by a wide range of environmental factors. Therefore, microbial consortia products (MCPs) based on multiple PGPM strains with complementary functions, have been proposed as superior, particularly under challenging environmental conditions and for restoration of beneficial microbial communities in disturbed soil environments. To test this hypothesis, the performance of a commercial MCP inoculant based on 22 PGPM strains was investigated in greenhouse experiments with maize on three soils with contrasting pH, organic matter content and microbial activity, under different P and N fertilization regimes. Interestingly, the MCP inoculant stimulated root and shoot growth and improved the acquisition of macronutrients only on a freshly collected field soil with high organic matter content, exclusively in combination with stabilized ammonium fertilization. This was associated with transiently increased expression of *AuxIAA5* in the root tissue, a gene responsive to exogenous auxin supply, suggesting root growth promotion by microbial auxin production as a major mode of action of the MCP inoculant. High microbial activity was indicated by intense expression of soil enzyme activities involved in C, N and P cycling in the rhizosphere (cellulase, leucine peptidase, alkaline and acid phosphatases) but without MCP effects. By contrast, the MCP inoculation did not affect maize biomass production or nutrient acquisition on soils with very little C_org_ and low microbial activity, although moderate stimulation of rhizosphere enzymes involved in N and P cycling was recorded. There was also no indication for MCP-induced solubilization of Ca-phosphates on a calcareous sub-soil fertilized with rock-phosphate. The results demonstrate that the combination of multiple PGPM strains with complementary properties as MCP inoculants does not necessarily translate into plant benefits in challenging environments. Thus, a better understanding of the conditions determining successful MCP application is mandatory.

## 1. Introduction

The adoption of biostimulants (BS) based on bacterial and fungal inoculants or non-microbial bioactive compounds (e.g., humic acids, amino acids and peptides, chitosan, plant-, seaweed-, and compost-extracts), has been discussed as a strategy to reduce the input of agrochemicals in crop production systems and related detrimental side effects on the environment [1,2]. Microbial and non-microbial BS may contribute to the mobilization of sparingly-soluble mineral nutrients, stimulate mineralization and nutrient cycling in the rhizosphere, promote root growth, and induce metabolic priming effects against biotic and abiotic stress factors in the target crops [3,4]. However, high variability and frequently limited reproducibility of the expected effects under real production conditions [5,6] suggests a strong impact of external factors, such as timing, dosage and mode of application, soil properties, fertilization management, environmental stress factors, interactions with the native soil microbiome, genotypic differences in responsiveness etc. To address this problem, the concept of consortia products based on different microbial strains and non-microbial BS with complementary properties was discussed as a strategy to increase the efficiency and the flexibility of BS-based production strategies under variable environmental conditions. Moreover, the composition of microbial consortia and non-microbial BS aims at the restoration of plant-beneficial, soil biological processes disturbed by soil degradation, intensive use of mineral fertilizers and chemical crop protection. This may apply for processes of nutrient cycling and mineralization, biological nitrogen fixation, nutrient mobilization and the pathogen suppressive potential in agricultural soils [4,7,8].

In this study, we aimed to characterize the performance of a commercial microbial consortia product (MCP) used as plant growth-promoting microorganism (PGPM) inoculant, based on carbon decomposers, providing easily available carbon sources for native rhizosphere biota and for microbial MCP strains involved in nutrient mineralization and biological N_2_ fixation. Together with the activities of P-, and K-solubilizing inoculant strains and root growth promoters, an improved nutritional status of the host plants and the related rhizosphere-microbial communities was expected. Chitinase producers, PGPM strains belonging to the genera *Bacillus*, *Paenibacillus, Pseudomonas* were included to promote pathogen antagonisms and induce priming effects against abiotic stress factors [9]. Based on this hypothetic scenario, the function of the MCP product was characterized under real rhizosphere conditions in greenhouse experiments, using maize as a model plant with a limited potential for root-induced nutrient mobilization [10]. The preliminary results indicated that the expression of MCP effects on plant growth and nutrient acquisition was influenced by the dosage of N and P fertilizers but also by the form of N supply. The most intense expression of MCP effects on plant growth was observed in combination with ammonium fertilization, stabilized with a nitrification inhibitor (3,4-dimethylpyrazolephosphate, DMPP) and reduced P availability, which was associated with increased root length development. However, MCP inoculant did not change activities of C-, N-, and P-cycling enzymes in the rhizosphere of MCP inoculated plants [11].

In addition to the characterization of MCP performance depending on N and P supply [11], the effect of different soil types with limited P availability was addressed in the present study. Three soils with contrasting properties were selected for the experiments. To test the hypothesis of preferential MCP performance in disturbed soil environments [4,9], experiment 1 compared MCP performance on two low P soils with moderate P fertilization. To include an active soil microbiome, a clay loam with high organic matter content was freshly collected from the Ap horizon of a field site after grassland conversion. To represent a heavily disturbed soil environment with low microbial activity, a 20-year-stored, air-dried sandy loam substrate, characterized by low organic matter content with a low pH buffering capacity was selected. A functional characterization of soil microbial activities in the respective soils was performed by recording marker enzyme activities involved in C, N and P cycling in the maize rhizosphere. In experiment 2, the P solubilizing potential of the MCP inoculant under rhizosphere conditions was tested on a low P, calcareous Loess subsoil with Ca-phosphates as major mineral P form and low organic matter content. Without additional application of soluble P fertilizers, P acquisition of maize on this soil substrate would be almost exclusively dependent on mobilization of Ca-P. In all experiments, nitrogen was applied in the form of nitrate or DMPP-stabilized ammonium. Plant biomass production and nutritional status, root growth characteristics, expression of auxin-responsive genes in the root tissue, as well modifications in rhizosphere chemistry (rhizosphere pH, marker enzyme activities involved in rhizosphere C, N and P cycling) were recorded to assess the effects of the MCP inoculants.

## 2. Materials and Methods

### 2.1. Soil Properties

Experiments were carried out on three different soils with contrasting properties. Soil 1 was a sandy-loam (pH_CaCl2_ 6.1) with low phosphate (P) availability (7 mg P_CAL_ kg^−1^, (12 VFLUFA, 1991)) with low organic matter content (C_org_ 0.58%) and low microbial activity due to approximately 20-years storage under air dried conditions. By contrast, Soil 2 was a freshly-collected clay-loam field soil (pH 5.9) with limited P availability (20 mg P_CAL_ kg^−1^) and high organic matter content (C_org_ 2.24%), to include an active soil microflora. The top soil was collected from the 0–30 cm Ap horizon at this field site. Soil 3 was a calcareous Loess sub-soil (pH 7.6) with low P availability (5 mg P_CAL_ kg^−1^) and low organic matter content (0.16%), supplied with rock-phosphate (Rock-P) fertilization to assess the potential of the MCP inoculant for mobilization of sparingly soluble Ca-P in the rhizosphere. All the soils were air-dried, sieved with 2 mm mesh size and mixed with 30% (*w/w*) quartz sand to improve the soil structure. The specific chemical and physical soil properties are listed in Table S1.

### 2.2. Fertilization

Fertilization of the substrates was adapted according to soil properties, the experimental questions and the duration of the experiments. For soils 1 and 2, macronutrient supply (mg kg^−1^ soil) comprised: N: 140 as calcium nitrate (Yara Liva Calcinit, Yara International, Oslo, Norway) or DMPP-stabilized ammonium sulfate (NovaTec^®^ Solub 21 (Compo Expert, Münster, Germany, with DMPP = 3,4-dimethylpyrazole phosphate as nitrification inhibitor), K: 150 as K_2_SO_4_; Mg: 50 as MgSO_4_, and a moderate soluble P fertilization with P: 30 as Ca(H_2_PO_4_)_2_. Soil 3 was fertilized with N: 100 as DMPP-stabilized ammonium sulfate (80%) and calcium nitrate (20%) to support P solubilization by ammonium-induced rhizosphere acidification [12]; K: 150 as K_2_SO_4_ and Mg: 50 as MgSO_4_. Phosphate (P: 80) was supplied as sparingly-soluble Rock-P (Granuphos; 18% P_2_O_5_; Landor, Birsfelden, Switzerland) or as soluble Ca(H_2_PO_4_)_2_ as a positive control treatment.

### 2.3. Test Plant and Culture Conditions

Maize (*Zea mays* L.) cv. Jessy (Advanta, Limagrain, Edemissen, Germany) was used as a test plant for all the experiments. Three seeds per pot were sown at depth of 1 cm and the soil surface was covered with a layer of fine quartz sand to minimize surface evaporation. For all experiments, the seeds originated from the same seed lot to account for potential differences in seed quality and the seed microbiome. After germination, thinning to one seedling per pot was performed and plant culture was conducted under greenhouse conditions with average air temperatures between 21 and 29 °C and 35–50% rel. humidity. Depending on the duration of the experiments between 28 and 41 days, substrate application comprised 2.4 kg (Soil 1), 2.9 kg (Soil 2) and 1.4 kg (Soil 3) per pot. Soil moisture was adjusted daily to 70% of the substrate water-holding capacity by gravimetric determination.

### 2.4. MCP Inoculation

The patented MCP inoculant [9] used in the experiment was provided by EuroChem Agro, (Mannheim, Germany) as a modified liquid formulation with 22 beneficial bacterial strains, including *Azotobacter vinelandii; Acetobacter pasteurianus; Bacillus amyloliquefaciens; Bacillus flexus; Bacillus licheniformis; Bacillus megaterium; Bacillus sp.; Bacillus subtilis; Clostridium beijerinckii; Clostridium pasteurianum; Lactobacillus casei/paracasei; Lactobacillus buchneri; Lactobacillus delbrueckii; Lactobacillus vini; Oceanobacillus oncorhynchi; Paenibacillus chibensis; Paenibacillus cookii; Paenibacillus lautus; Pseudomonas sp.; Pseudomonas putida; Streptomyces griseus; Virgibacillus halophilus* as declared ingredients. For inoculation, a suspension of MCP 0.01325% (*w*/*w*) with non-chlorinated tap water was applied by soil-drenching close to the stems of plants (10 mL per plant for each inoculation step) depending on the duration of the experiments at 0, 14, 28 days after sowing (DAS) on Soil 1; 0, 7, 21, 34 DAS on Soil 2 and 0, 14, 21 and 34 DAS on Soil 3. Control plants (Ctrl) were treated with the respective amounts of non-chlorinated tap water.

### 2.5. Plant Growth and Nutritional Status

At final harvest, the root systems were washed out of the soil substrate, and loosely adhering rhizosphere soil was collected by shaking and stored at −20 °C until further analysis. Root length was determined after digitalization using the WinRhizo root analysis system (Regent Intruments, Quebec Canada) and root and shoot dry matter were determined gravimetrically after oven-drying at 60 °C. For analysis of the plant nutritional status, 250 mg of dried shoot material was subjected to 1.5 h microwave digestion at 1400 W (ETHOSlab Professional Microwave System, MLS, Leutkirch, Germany) after 30 min extraction in 5 mL HNO_3_ (conc.) 1:3, 3 mL H_2_O_2_ (30%) and 2 mL deionized water. Spectrophotometric determination of orthophosphate was conducted after addition of molybdate-vanadate color reagent according to [13] using a Hitachi U-3300 Spectrophotometer, Hitachi Ltd., Tokyo, Japan). The concentrations of K and Ca were determined with an ELEX 6361 flame-photometer, Eppendorf, Germany). The concentrations of Mg were measured with an iCE 3000 Series Atomic Absorption Spectrometer (ThermoScientific, USA). Total shoot N was analysed with a Vario Max CN macro-elementar analyser (Elementar Analysensysteme, Hanau, Germany). N_min_ analysis of the bulk soil was conducted with 20 g samples of previously cold-stored (4 °C) soil, mixed with 80 mL of 0.0125 M CaCl_2_ solution in a shaker for 1 h 200 rpm. After settling of the soil particles, the supernatant was filtered with 617 ¼ pleated filters (Machery-Nagel, Düren, Germany), The filtrates were stored in plastic bottles at −20 °C until analysis. The N_min_ analysis of the soil solutions was conducted with an AutoAnalyzer 3 (SEAL Analytical, Southampton, UK) for NO_3_^−^-N and NH_4_^+^-N, respectively.

### 2.6. Expression of Auxin-Responsive Genes in the Root Tissue

The expression of auxin-responsive genes was tested using total RNA extracted from washed maize roots. After harvest roots were directly frozen in −80 °C, homogenized in liquid N_2_ and total RNA was extracted using the innuPREP plant RNA Kit (Analytic Jena, Jena, Germany) following the manufactures instruction. Using the Quanti Tect Reverse Transcription Kit (Quiagen, Hilden, Germany), 1 µg of RNA was reverse transcribed into cDNA. Quantitative real time PCR for the auxin related genes IAA5 and PIN1c was performed using 15 ng cDNA per reaction. The reaction was performed in a CFX384 (Bio-Rad, Hercules, CA, USA) using the GreenMasterMix (2×) without ROX (Genaxxon bioscience GmbH, Ulm, Germany). Data were analyzed using the Bio-Rad CFX Manager 3.1 software. The housekeeping genes EF1a as well as beta-Tubulin were used as reference genes to normalize the expression data. Technical replicates were performed in triplicate. Expression of PIN1c was tested in one qPCR using three technical repetitions for two biological replicates.

### 2.7. Marker Enzymes as Indicators for C, N and P Cycling in the Rhizosphere

The activities of marker enzymes for C, N and P cycling in the rhizosphere soil was assayed with fluorogenic substrates containing the fluorescence indicator 4-methylumblliferon (4-MUF; Sigma-Aldrich, St. Louis, MO, USA) according to the method described by Stemmer [14]. A microplate reader (Microplate Fluorescence reader FL × 800, BioTek Instruments Inc., Winooski, VT, USA) was used for monitoring the enzymatic hydrolysis of the MUF substrates for L-leucin peptidase (EC 3.4.11.1), cellulase (EC 3.2.1.21), acid (EC 3.1.3.2) and alkaline phosphomonosterase (EC 3.1.3.1) at 360/460 nm.

### 2.8. Experimental Design and Statistical Evaluation

All experiments were arranged in a completely randomized design with five replicates and each one plant per treatment (Soil 1 and 2). Due to the extremely low P availability, the experiment on soil 3 comprised 10 replicates per treatment, since a weaker expression of MCP effects was expected under these conditions.

Statistical analysis was performed with SAS^®^ 9.4 (SAS Institute Inc., Cary, NC, USA) statistical software by one-way ANOVA with Tukey test *p* ≤ 0.05 for testing significant differences between treatments. Outliers were identified and removed based on the quartile method of [15].

An overview with a compilation of all experimental treatments is presented in Table S2.

## 3. Results

In the first experiment, potential MCP effects were compared on two moderately acidic low P soils with comparable pH (≈ 6) but contrasting properties with respect to soil texture, organic matter content and microbial activity. A first harvest was conducted between week 4 and 5 after sowing, when the first effects of the MCP inoculation became visually detectable (Figure S1).

### 3.1. Plant Growth

**Figure 1:**
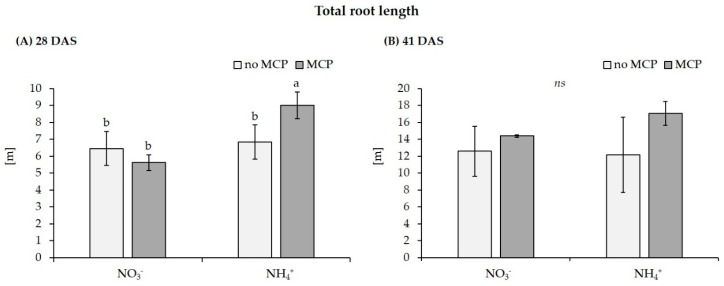
Effect of MCP inoculation on total root length of maize (cv Jessy) after a culture period of (**A**) 28 DAS and (**B**) 41 DAS on a clay-loam field soil (pH 5.9) with low P availability (Soil 2) supplied with moderate soluble P fertilization (30 mg P kg^−1^) and N in form of Ca-nitrate (NO_3_^−^) or DMPP-stabilized ammonium (NH_4_^+^) Data represent means and SD of five replicates. A one-way ANOVA with Tukey test was performed. Different letters indicate significant differences between treatments (*p* < 0.05); ns = not significant.

After a culture period of 4–5 weeks, shoot growth was superior on the sandy loam soil with low C_org_ and limited microbial activity, despite massively reduced root length compared to Soil 2. Furthermore, shoot accumulation of P was approximately doubled on Soil 1 and significantly increased in response to stabilized ammonium fertilization versus nitrate supply. However, root length even declined by approximately 60% in the variants with ammonium fertilization and MCP inoculation was entirely without effect on Soil 1 (Table 1, Figure 1).

By contrast, on the freshly collected clay-loam field soil (Soil 2), only the combination of stabilized ammonium fertilization with MCP inoculation significantly increased shoot biomass by 29% compared with the non-inoculated control with ammonium supply and even by 63% relative to nitrate fertilization. This was associated with a significantly increased root length by 32% and increased P accumulation (34%) exclusively in the MCP-ammonium combination.

### 3.2. Rhizosphere Chemistry

On both soils, the rhizosphere pH was higher with nitrate fertilization than with stabilized ammonium supply. The ammonium-induced rhizosphere acidification was particularly intense on the sandy loam (Soil 1), and reached a pH range between 4.6 and 5.1, indicating a low pH buffering capacity of the sandy substrate (Table 2).

Rhizosphere marker enzyme activities for C, N and P cycling were significantly higher on the freshly collected clay loam field soil as compared with the sandy loam (factor 4–16). This effect was particularly expressed for alkaline and acid phosphatase activities. As expected, on the clay loam with high endogenous microbial activity, MCP inoculation had no significant additional effects on enzyme activities. However, MCP application stimulated leucine peptidase activity by 36%, acid phosphatase by 39% and alkaline phosphatase by 28% on the sandy loam in combination with ammonium fertilization, while with nitrate fertilization a significant increase by 12% was recorded only for the activity of acid phosphatase (Table 2).

### 3.3. Plant-Nutritional Status

On both soils, shoot P concentrations dropped below the sufficiency threshold of 3 g kg^−1^ DM [16] (Table 3). However, analysis of shoot P accumulation revealed an increase by 27–34% induced by stabilized ammonium fertilization in comparison with nitrate supply (Table 4). Similar to the stimulation of root growth, a MCP effect on shoot P accumulation was exclusively detectable on the clay loam soil in combination with stabilized ammonium supply, with an increase by 34% compared with the non-inoculated control and even 71% compared with nitrate fertilization (Table 4).

On both investigated soils, shoot concentrations of N were low, but still in the sufficiency range (deficiency threshold 30 g N kg^−1^ DM) and the K status was sufficient (Table 3). Similar to the shoot P content, also N and K accumulation was significantly increased by 30% and 33%, respectively after MCP inoculation on the fresh clay loam field soil with stabilized ammonium fertilization (Table 4).

Shoot Ca and Mg concentrations were reduced under ammonium fertilization on both soils, as compared with nitrate supply and reached critical values close to the deficiency thresholds on the sandy loam soil (Table 3). Again, MCP inoculation induced a significantly increased shoot accumulation of Ca and Mg by 24 and 23%, exclusively on the silty loam soil with stabilized ammonium fertilization (Table 4).

### 3.4. MCP Effects on Root Growth and Expression Auf Auxin-Responsive Genes in the Root Tissue

The selective MCP-induced promotion of root growth on the clay loam field soil with stabilized ammonium fertilization was investigated in more detail. Root growth promotion by MCP and bacterial single strain inoculants in combination with stabilized ammonium supply have been similarly reported in recent studies and were associated with increased auxin production potential of bacterial populations re-isolated from the rhizosphere of inoculated maize plants [11,17]. This may pinpoint to root growth stimulation based on microbial auxin production.

As a more direct indicator, in this study, we investigated the expression of selected auxin-responsive genes in the root tissue of MCP-inoculated maize plants in relation to root growth responses. The *ZmAuxIAA5* gene was selected as a well-studied member of the auxin early response genes of the Auxin/Indole-3-Acetic Acid (Aux/IAA) family, that is rapidly up-regulated by external auxin supply [18]. The *ZmPIN1c* gene encodes an auxin efflux transporter involved in shoot to root translocation of auxins [19] and was selected as potential indicator of MCP effects on internal plant auxin homeostasis, independent of microbial auxin production. To address the longevity of the effects, measurements were conducted at two time points at 28 DAS and 41 DAS. For both time points, harvest was performed approximately one week after the last MCP inoculation.

**Figure 2:**
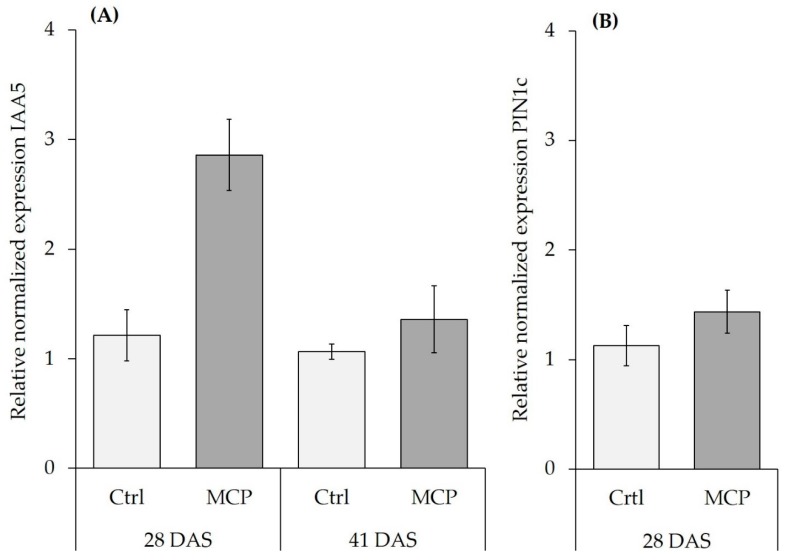
Relative normalized expression of auxin-related genes *ZmAux IAA5* (IAA5) and Zm*PIN1c* (PIN1c) in root tissue of control (Ctrl) and MCP inoculated (MC) maize plants grown on a clay-loam field soil (pH 5.9) with low P availability (Soil 2) supplied with moderate soluble P fertilization (30 mg P kg^−1^) and N in form of DMPP-stabilized ammonium (NH_4_^+^). (**A**) IAA5 expression at 28 and 41 days after sowing (DAS). (**B)** PIN1c expression in plants at 28 DAS. Data for IAA5 represent means ± SD of two biological replicates, with nine technical replicates. For PIN1c data represent means ± SD of two biological replicates with three technical repetitions.

A selective MCP-induced stimulation of root length was detected at 28 DAS on the fresh clay loam field soil with stabilized ammonium supply, but this effect disappeared at 41 DAS, showing only a gradual, non-significant increase. This was associated with a significant increase of *ZmAuxIAA5* expression at 28 DAS, but not at 41 DAS, whereas the expression of *ZmPIN1c* remained unaffected (Figure 2). Since MCP-induced root growth promotion was dependent on ammonium supply, we measured N_min_ in the remaining clay loam after harvest. Nitrate was the dominant N form detected in all variants. Compared with the initial N_min_ level of 144 mg kg^−1^ substrate, at 28 DAS, N_min_ was depleted by 58% in the nitrate variant and by 71% in the ammonium variant, where only 0.3% of the total N_min_ applied by fertilization remained in the ammonium form. At 41 DAS, nitrogen was depleted by >95% from the pots in all variants. In general, depletion of N_min_ was faster in the MCP variants as compared with the non-inoculated control, particularly under stabilized ammonium fertilization. Interestingly, ammonium N increased between 21 and 41 DAS, except for the ammonium variant lacking MCP (Table 5).

### 3.5. Phosphate-Solubilizing Potential of the MCP Inoculant

Experiment 2 was designed to assess the solubilizing potential for sparingly soluble soil P forms of the MCP inoculant. A calcareous Loess subsoil pH 7.6 with 23% calcium carbonate and extremely low P availability (P_CAL_ 5 mg kg soil^−1^) and very low C_org_ was selected as culture substrate. Phosphate was applied as sparingly-soluble Rock-P or, with soluble Ca(H_2_PO_4_)_2_ as a positive control. In the Rock-P variants, plant P acquisition was only possible after solubilization of Ca-P. Stabilized ammonium sulfate was applied as major N form to exploit the beneficial effects of ammonium on plant-MCP interactions and of ammonium-induced rhizosphere acidification on P solubility, similarly to experiment 1.

**Figure 3:**
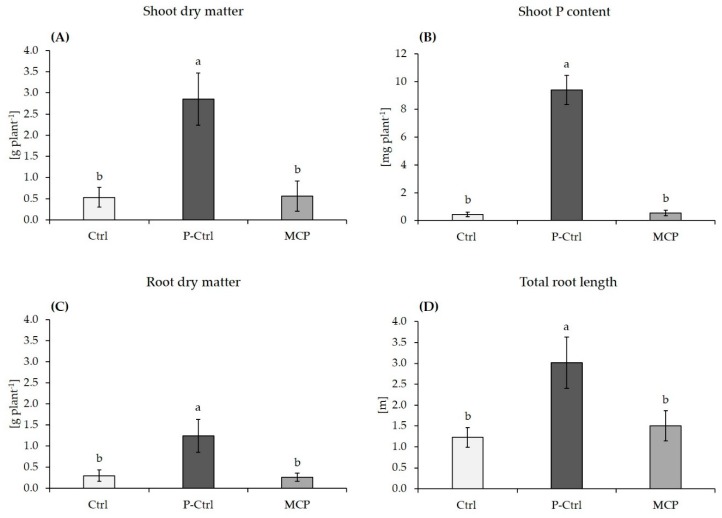
Shoot biomass (**A**), P content in shoot tissue (**B**), root dry matter (**C**) and total root length **D**)of maize plants (cv. Jessy) grown for 37 days on a calcareous Loess subsoil (pH 7.6) supplied with Rock-P (Ctrl, MCP treatment) or with soluble Ca(H_2_PO_4_)_2_ as positive control (P-Ctrl). All plants received DMPP-stabilized ammonium (80 mg N kg soil^−1^) and calcium nitrate (20 mg N kg soil^−1^) as nitrogen sources. Data represent means and SE of ten replicates. A One-way ANOVA with Tukey test was performed for data comparison. Different letters indicate significant differences between treatments (*p* < 0.05).

In the variants with Rock-P fertilization, MCP inoculation had no effect on shoot and root biomass production, root length and shoot P accumulation, as compared with the non-inoculated control (Figure 3A–D). Ammonium fertilization was not associated with rhizosphere acidification and the rhizosphere pH remained constant in all variants (pH 7.8). Although MCP inoculation had a marginal positive effect on the shoot P concentration, the P status remained severely deficient [16] and did not exceed 1 g kg^−1^ DM (Figure 3B). Only in the positive control with soluble P fertilization, the shoot P status reached the sufficiency range (3.3 g kg^−1^ DM), associated with an increase in shoot biomass by 450% (Figure 3A,B).

## 4. Discussion

### 4.1. Effects of N-Form Supply on MCP Performance on Different Soils

The results of the present study confirm a beneficial effect of stabilized ammonium fertilization on the establishment of plant growth-promoting interactions, induced by MCP and other PGPM inoculants in plants with limited P supply (Table 1), as also previously reported by [11,17,20]. Synergistic interactions between ammonium fertilization and PGPM inoculation have been attributed to a range of different factors: (i) Root-induced rhizosphere acidification via proton extrusion in response to preferential ammonium uptake, which mediates the solubilization of acid-soluble soil P fractions [21], providing a P starter supply to the host plant under P-limited conditions [17,20]. This effect was detectable even on the slightly acidic soils (≈ pH 6) investigated in this study, where P was identified as limiting nutrient (Table 3; Table S1). The improved P-nutritional status promoted the establishment of beneficial plant-PGPM interactions in the rhizosphere. Accordingly, impaired rhizosphere establishment of PGPMs in extremely P deficient plants is well-documented [20,22,23,24]; (ii) Root growth promotion induced by the MCP inoculant has been observed in this study on the clay loam soil (Table 1). This improves not only P acquisition, but nutrient uptake in general (Table 4); [20,24]; (iii) ammonium fertilization can stimulate root hair development [17,25,26]; and (iv) ammonium nutrition stimulated auxin production of maize plants and various bacterial PGPM strains belonging to the genera *Bacillus*, *Pseudomonas* and *Acetobacter* [17,27,28]. Accordingly, also Bradacova et al. 2019 [11] found increased auxin production of bacterial populations that were re-isolated from the rhizosphere of MCP-inoculated maize plants with stabilized ammonium supply. Strains of *Bacillus*, *Pseudomonas* and *Acetobacter* are also part of the investigated MCP formulation and therefore may be similarly involved in the increased auxin production of the bacterial populations in the maize rhizosphere after MCP inoculation [11].

Root growth stimulation via additional auxin supply by PGPM inoculants has been discussed as a major mechanism for plant growth promotion via microbes [29]. However, similar growth promoting effects have been demonstrated for bacterial quorum sensing signals or various volatile organic compounds (VOC′s) of fungal and bacterial origin, which may interfere with auxin homeostasis in plants [29,30,31]. *AuxIAA5* gene expression is known to be rapidly induced by external auxin supply in roots [18]. In our study, *AuxIAA5* expression was upregulated on the silty loam field soil with ammonium fertilization one week after the last inoculation with MCP. This effect was restricted to 28 DAS, when increased root growth was apparent but was not detectable at 41 DAS. By contrast, expression of the *PIN1c* gene, that encodes an IAA efflux transporter, was not affected by MCP, supporting the hypothesis that the MCP-inoculant, but not the plant itself, supplied increased auxin levels to the root [11] *PIN1c* is activated by shoot-borne IAA and potentially involved in adventious rooting and acropetal auxin transport in the central cylinder [32]. By contrast, Garnica-Vergara 2016, [33] found an auxin-independent activation of *PIN* genes (*PIN1, PIN2, PIN3, PIN7*), associated with increased lateral root formation in *Arabidopsis thaliana* by 6-pentyl-2H-pyran-2-one (6-PP), a major bioactive VOC with potential cross-kingdom signaling functions emitted by *Trichoderma spp.* However, *Trichoderma* strains were not included in the MCP formulation tested in this study and accordingly PIN1c expression remained unaffected by MCP inoculation. Apart from root growth promotion, MCP inoculation also exerted stimulatory effects on rhizosphere marker enzyme activities for processes involved in mineralization of N (leucine peptidase) and P (acid and alkaline phosphatases), mainly in combination with stabilized ammonium fertilization (Table 2). In accordance with the hypothesis that MCP applications are particularly effective in disturbed environments by stimulating the recovery of beneficial microbial functions, positively linked to soil fertility [4,9], MCP effects on rhizosphere peptidases and phosphatases were detected exclusively on the sandy-loam soil that was stored for 20 years under air-dried conditions (Table 2) with very low C_org_ and limited microbial activity. In this soil, MCP inoculation stimulated peptidase and phosphatase activities, but the activity level still remained lower by magnitudes than in the freshly-collected clay loam field soil with an active soil microbiome (factor 4–16; Table 2). This underlines the generally low microbial activity in the sandy loam soil. Interestingly, higher peptidase and phosphatase activities in the rhizosphere of the MCP-inoculated maize plants in the sandy loam soil did not translate into any improvement of nutrient acquisition (Table 3 and Table 4) and plant growth (Table 1). Substrate limitation for the enzymes due to the low organic matter content of the sandy loam soil (0.58%) may offer a possible explanation. In this context, co-application of organic fertilizers with MCP inoculants could offer a promising alternative, as recently shown for various single strain inoculants in combination with composted manures on low P soils in maize [23,34,35] and tomato [24]. By contrast, on the freshly-collected clay loam field soil, high background activities of cellulases, peptidases and phosphatases, reflecting a pronounced indigenous microbial activity, may have masked the comparatively small effects induced by the MCP-inoculants (Table 2).

The weak expression of MCP effects on marker enzyme activities involved in C, N and P cycling in the rhizosphere also indicates a limited direct or indirect impact of the MCP inoculation on these processes, e.g., by interactions with the soil microbiome. By contrast, a recent follow-up study demonstrated improved P acquisition of tomato after MCP inoculation combined with stabilized ammonium fertilization, in a drip-irrigated field experiment conducted in the Negev desert in Israel. Under these conditions, significant microbiome effects were detectable even three months after the last MCP inoculation [36]. An increased bacterial alpha-diversity at the rhizoplane was associated with a reduced abundance of *Sphingobacteria* known as salinity indicators and an increase in the population density of potentially plant-growth-promoting *Flavobacteria*. However, also in this case it was not clear whether these effects must be regarded as a cause or rather as a consequence of the improved P status of the host plants, induced by MCP inoculation [36].

### 4.2. Limitations of Combined MCP Application with Ammonium Fertilizers

The beneficial MCP effects on plant growth were significantly promoted by combined application with stabilized ammonium fertilization (Table 1; [11]), but rapid microbial transformation of ammonium to nitrate in well-aerated agricultural soils may impede the practical use of this observation in agronomy. Therefore, the use of nitrification inhibitors seems to be mandatory to counteract rapid N turnover. However, nitrification inhibitors, such as DMPP, are active in soils only for limited time periods due to microbial degradation [37]. This explains the almost complete nitrification of ammonium already at 28 DAS on the clay loam soil. Accordingly, root growth promoting effects of MCP inoculation (Figure 1) and related upregulation of the auxin-responsive *AuxIAA5* gene in the root tissue of the maize plants (Figure 2) disappeared with increasing duration of the experiment at 41 DAS, even after repeated MCP applications. Longer-lasting effects of nitrification inhibitors may be expected under field conditions due to lower soil temperatures, lower rooting densities, and reduced rhizosphere microbial activities as compared with pot experiments. However, also under field conditions, plant growth-promoting effects of single strain inoculants and microbial consortia were most intensively expressed during early growth of maize plants [20]. Efficient field establishment is a critical factor for yield formation of maize particularly under unfavorable environmental conditions [38,39], but the establishment of longer lasting PGPM effects in agricultural production systems still remains a major challenge. Apart from limited stability of nitrification inhibitors in natural environments, repeated PGPM inoculations close to the root system for an efficient long-lasting root colonization are technically and economically challenging. This may be less difficult in horticultural production, were repeated application of PGPMs and stabilized ammonium fertilizers close to the roots is more easily integrated into widely-used drip irrigation or fertigation systems.

Apart from limited longevity, the expression of MCP effects was evidently also influenced by the respective soil properties. On the two moderately acidic soils (≈ pH 6), plant-growth promoting effects after MCP application were exclusively detectable on the fresh clay loam field soil with stabilized ammonium fertilization. However, the high endogenous microbial activities in this soil, reflected by high activities of rhizosphere enzymes, were not affected by the inoculation. As expected, increased rhizosphere enzyme activities involved in N and P cycling were detectable only in the MCP-inoculated variants of the sandy loam soil with a low intrinsic microbial activity but this did not translate into promotion of plant growth. By contrast, on the fresh silty loam field soil, MCP inoculation promoted root length development that resulted in a generally improved acquisition of macronutrients. An improved P-nutritional status could be partially attributed to increased P-solubilization due to moderate ammonium-induced rhizosphere acidification to pH 5.3–5.4 (Table 2). This was further supported by the root growth-promoting effects of the MCP inoculant (Table 1), leading to a larger acidifying root system for P mobilization and uptake. Improved P shoot accumulation due to ammonium-induced rhizosphere acidification was also detectable on the sandy-loam soil. However, due to a low pH-buffering capacity of the substrate, the rhizosphere pH in the ammonium variant dropped to pH 4.6 (Table 2), associated with inhibitory effects on root growth (Table 1). This was reflected in a 60% reduction in root length as compared with nitrate fertilization. At soil pH levels below 5.0, the risk of root growth inhibition induced by aluminium toxicity increases [40]. Moreover, the plant Ca and Mg nutritional status declined close to the critical thresholds (Table 3) as a consequence of the well-documented antagonistic effects of ammonium nutrition on uptake of cations, such as Ca and Mg [41]. This can lead to induced Ca and Mg deficiencies with detrimental effects on root growth [42,43,44]. Under these adverse conditions for root development, the root-growth promoting potential of the MCP inoculant was obviously not sufficient to exert any stimulatory effects on root growth and nutrient acquisition due to detrimental effects of limited root development on rhizosphere establishment of the inoculant.

By contrast, on the highly buffered and slightly alkaline subsoil (pH 7.6) with 23% calcium carbonate that was supplemented with Rock-P (experiment 2), ammonium fertilization failed to induce rhizosphere acidification, and the rhizosphere pH remained at pH 7.8 in all treatments. Phosphate was identified as growth-limiting factor, indicated by a 450% increased shoot biomass production and a P-nutritional status in the sufficiency range after application of soluble Ca(H_2_PO_4_)_2_ instead of Rock-Phosphate as P fertilizer (Figure 3). However, even in combination with ammonium fertilization, the MCP inoculation had no beneficial effects on shoot biomass, root length development and P acquisition as compared with the non-inoculated control (Figure 3), and the plant P-nutritional status was severely deficient [16].These findings demonstrate that under the selected conditions, neither ammonium fertilization, nor MCP inoculation had any effects on solubilization of sparingly soluble Ca-phosphates, probably due to the high pH buffering capacity of the calcareous soil substrate. Moreover, similarly to the sandy loam soil, root development was severely inhibited in the severely P-deficient plants (Figure 3) as limiting factor for MCP root colonization. Instead the placement of homogenous application of the ammonium fertilizer may offer a solution to overcome this problem. Jing et al. [45] demonstrated that localized root proliferation in response to ammonium placement resulted in a significant decrease of rhizosphere pH of field-grown maize plants by two units even on alkaline soils with pH 8 in the ammonium depot zone. This effect was not detectable after broadcast application of ammonium fertilizers and was attributed to the high density of acidifying lateral roots in the application zone, increasing the intensity of rhizosphere acidification. This finally translated into improved P mobilization during early growth. Moreover, Nkebiwe et al. [46] demonstrated increased ammonium-induced root proliferation in the placement zone after PGPM inoculation of field-grown maize plants. These observations suggest that the combination of ammonium placement with PGPM inoculation could offer a strategy to overcome limitations of ammonium-induced P solubilization, otherwise limited by the high pH buffering capacity of the substrate.

### 4.3. Concluding Remarks

Based on the results of the present study, the beneficial effects of the selected MCP inoculant on plant growth and nutrient acquisition were strongly dependent on the form of nitrogen fertilization, soil properties and the plant developmental stage, as has been previously shown also for various single-strain inoculants [17,20,24]. Contrary to the initial hypothesis, compensating functions and preferential performance of the MCP inoculants in terms of plant growth promotion were not recorded on disturbed soils with limited nutrient availability, low microbial activity and low C_org_. By contrast, MCP effects were preferentially expressed on a freshly-collected silty loam field soil with abundant, active microbes and moderate P availability.

Among the various modes of action discussed for beneficial PGPM effects on plant growth, stimulation of root growth, probably mediated via microbial auxin production, was identified as a major feature of the selected MCP inoculant. By contrast, stress factors, such as extreme P limitation (Figure 1) or soil acidity (Table 2) with inhibitory effects on root growth, already during the sensitive phase of MCP rhizosphere establishment, were associated with a limited expression of beneficial MCP effects on plant growth (Table 1; Figure 1). Under these conditions even multiple inoculant strains with differences in stress tolerance only have a limited advantage, as long as the stress conditions affect the ability of the host plant to support the establishment of a functional MCP interaction in the rhizosphere. Since this scenario is more likely in agricultural crops directly sown under field conditions as compared with greenhouse or nursery cultures, it remains a major challenge for practical applications. Although stimulatory effects of the inoculant on N and P cycling in the rhizosphere were detectable, these were not sufficient to translate into any benefits for plant nutrient acquisition or growth. Overall, there was no indication for solubilization of Ca-phosphates by the inoculant that could support plant P acquisition.

The findings of the present study demonstrated that similarly to single strain inoculants, MCP inoculants do not have a universal plant-growth promoting functions, but their successful application strongly depends on various external factors. A better knowledge of the exact conditions required for the induction of beneficial effects on plant growth could provide a perspective for a more directed application of MCP-assisted production strategies.

## Figures and Tables

**Table 1:** Shoot dry weight, total root length and P content in shoot tissue of maize plants (cv. Jessy) on two low P soils. as affected by MCP inoculation. Soil 1 (sandy-loam, pH 6.1, P_CAL_ 7 mg kg^−1^ soil) and Soil 2 (clay-loam, pH 5.9, P_CAL_ 20 mg kg^−1^ soil) receiving N fertilization in in form of Ca-nitrate (NO_3_^−^) or DMPP-stabilized ammonium (NH_4_^+^) and a moderate soluble P supply (30 mg P kg^−1^). Data represent means and SD of five replicates. A one-way ANOVA with Tukey test was performed for data comparison. Different letters indicate significant differences between treatments (*p* < 0.05).

Plant Response	MCP Treatments	Soil 1		Soil 2	
		NO_3_^−^	NH_4_^+^	NO_3_^−^	NH_4_^+^
Shoot DW [g]	no MCP	3.32 ± 0.3 a	3.96 ± 0.2 a	2.09 ± 0.4 b	2.63 ± 0.3 b
	with MCP	3.37 ± 0.1 ab	3.90 ± 0.2 a	2.28 ± 0.5 b	3.4 ± 0.3 a
Total root length [cm]	no MCP	2718.9 ± 787.0 a	1048.3 ± 170.2 b	6454.6 ± 2954.1 b	6836.5 ± 4455.3 b
	with MCP	2325.3 ± 232.9 a	988.9 ± 448.6 b	5615.1 ± 132.0 b	9008.4 ± 1409.8 a
P content [mg plant^−1^]	no MCP	6.05 ± 0.8 b	8.10 ± 0.4 a	2.89 ± 0.5 b	3.67 ± 0.4 b
	with MCP	6.50 ± 0.4 b	7.60 ± 0.6 a	2.95 ± 0.8 b	4.93 ± 0.4 a

**Table 2:** Rhizosphere pH and rhizosphere-enzymatic activities of cellulase, peptidase and phosphatases as affected by MCP inoculation on soil 1 (sandy-loam, pH 6.1 P_CAL_ 7 mg kg^−1^ soil) and soil 2 (clay-loam, pH 5.9, P_CAL_ 20 mg kg^−1^ soil) receiving N fertilization in form of Ca-nitrate (NO_3_^−^) or DMPP-stabilized ammonium (NH_4_^+^) and a moderate soluble P supply (30 mg P kg^−1^). Data represent means and SD of five replicates. A one-way ANOVA with Tukey test was performed for data comparison. Different letters indicate significant differences between treatments (*p* < 0.05). * indicates a significant difference between MCP inoculated (with MCP) and non-inoculated (no MCP) plants within the same N fertilizer treatments (Tukey test at *p* < 0.05).

	MCP Treatments	Soil 1		Soil 2	
		NO_3_^−^	NH_4_^+^	NO_3_^−^	NH_4_^+^
	no MCP	5.11 ± 0.1 a	4.60 b ± 0.1	5.79 ± 0.02 a	5.35 ± 0.04 b
	With MCP	5.42 ± 0.4 a	5.11 ± 0.2 a	5.79 ± 0.01 a	5.32 ± 0.06 b
Rhizosphere Enzymatic Activities [nmol g^−1^ soil h^−1^]					
Peptidase	no MCP	42.01 ± 10.9	36.34 ± 6.2	160.28 ± 12.2	140.81 ± 6.6
	with MCP	47.92 ± 0.3	49.54 ± 1.6 *	142.60 ± 6.8	144.42 ± 11.9
Cellulase	no MCP	8.49 ± 0.9	7.67 ± 0.7	51.34 ± 5.5	52.28 ± 4.2
	with MCP	9.74 ± 1.0	8.15 ± 1.1	52.10 ± 1.1	48.19 ± 6.6
Acid Phosphatase	no MCP	111.76 ± 7.1	131.78 ± 22.2	964.96 ± 128.1	891.49 ± 28.0
	with MCP	125.62 ± 4.3 *	182.89 ± 19.5 *	948.89 ± 45.2	934.45 ± 125.2
Alkaline Phosphatase	no MCP	12.45 ± 3.6	7.0 ± 0.5	122.56 ± 18.8	113.96 ± 17.2
	with MCP	13.15 ± 2.3	8.98 ± 1.1 *	113.66 ± 17.3	127.16 ± 23.4

**Table 3:** Shoot concentrations of mineral nutrients of maize plants (cv. Jessy) on two low P soils. as affected by MCP inoculation. Soil 1 (sandy-loam, pH 6.1, P_CAL_ 7 mg kg^−1^ soil) and Soil 2 (clay-loam, pH 5.9, P_CAL_ 20 mg kg^−1^ soil) receiving N fertilization in in form of Ca-nitrate (NO_3_^−^) or DMPP-stabilized ammonium (NH_4_^+^) and a moderate soluble P supply (30 mg P kg^−1^). Data represent means of five replicates. A one-way ANOVA with Tukey test was performed for data comparison. Different letters indicate significant differences between treatments (*p* < 0.05).

Shoot Mineral Concentration (g kg DM^−1^)							
	N Forms	MCP Treatments	N	P	K	Ca	Mg
Soil 1	NO_3_^−^	no MCP	26.21 a	1.87 bc	51.19 a	3.94 a	1.99 a
		with MCP	25.25 a	1.82 c	49.22 a	3.95 a	1.99 a
	NH_4_^+^	no MCP	26.75 a	2.10 a	50.38 a	2.73 b	1.82 b
		with MCP	27.37 a	2.01 ab	49.55 a	2.75 b	1.74 b
Soil 2	NO_3_^−^	no MCP	37.54 a	1.38 a	36.75 b	4.92 a	2.71 a
		with MCP	35.42 b	1.24 b	36.61 b	4.81 a	2.65 a
	NH_4_^+^	no MCP	37.50 a	1.42 a	38.60 ab	4.15 b	2.33 b
		with MCP	37.73 a	1.45 a	39.88 a	4.00 b	2.12 c
Deficiency Threshold [16]			30.00	3.00	20.00	2.50	1.50

**Table 4:** Shoot accumulation of mineral nutrients in maize plants (cv. Jessy) on two low P soils. as affected by MCP inoculation. Soil 1 (sandy-loam, pH 6.1, P_CAL_ 7 mg kg^−1^ soil) and Soil 2 (clay-loam, pH 5.9, P_CAL_ 20 mg kg^−1^ soil) receiving N fertilization in in form of Ca-nitrate (NO_3_^−^) or DMPP-stabilized ammonium (NH_4_^+^) and a moderate soluble P supply (30 mg P kg^−1^). Data represent means of five replicates. A One-way ANOVA with Tukey test was performed for data comparison. Different letters indicate significant differences between treatments (*p* < 0.05).

Shoot Mineral Content (mg Plant^−1^)							
	N Form	MCP Treatments	N	P	K	Ca	Mg
Soil 1	NO_3_^−^	no MCP	88.62 a	6.05 b	163.7 b	12.67 ab	6.42 a
		with MCP	90.96 a	6.54 b	177.3 ab	14.60 a	7.28 a
	NH_4_^+^	no MCP	104.7 a	8.13 a	195.9 a	10.57 b	6.87 a
		with MCP	103.2 a	7.59 a	186.9 a	10.40 b	6.87 a
Soil 2	NO_3_^−^	no MCP	78.10 b	2.89 b	76.66 b	10.23 b	5.40 a
		with MCP	80.64 b	2.95 b	83.79 b	10.98 ab	5.84 a
	NH_4_^+^	no MCP	98.67 b	3.67 b	101.7 b	10.93 ab	5.87 a
		with MCP	128.1 a	4.93 a	135.5 a	13.57 a	7.21 a

**Table 5:** Effect of MCP inoculation on total N_min_, NO_3_-N [mg kg^−1^ soil DM] and NH_4_^+^_−_N [mg kg^−1^ soil DM] after a culture period of 28 DAS and 41 DAS on a clay-loam field soil (pH 5.9) with low P availability (Soil 2) supplied with moderate soluble P fertilization (30 mg P kg^−1^) and N in form of Ca-nitrate (NO_3_^−^) or DMPP-stabilized ammonium (NH_4_^+^) Data represent means and SD of five replicates. A one-way ANOVA with Tukey test was performed. Different letters indicate significant differences between treatments (*p* < 0.05).

	N Form	MCP Treatments	N_min_ Total	Soil NO_3_^−^-N	Soil NH_4_^+^-N
28 DAS	NO_3_^−^	no MCP	60.45 ± 5.1a	60.45 ± 5.1 a	0 ± 0 c
		with MCP	45.46 ± 2.8 b	45.46 ± 2.8 b	0 ± 0 c
	NH_4_^+^	no MCP	42.58 ± 4.1 b	42.12 ± 4.1 b	0.46 ± 0.05 a
		with MCP	19.34 ± 0.5 c	19.26 ± 0.4 c	0.08 ± 0.04 b
41 DAS	NO_3_^−^	no MCP	2.73 ± 1.0 b	2.67 ± 0.9 bc	0.06 ± 0.02c
		with MCP	3.72 ± 4.4 b	3.69 ± 4.4 b	0.06 ± 0.04c
	NH_4_^+^	no MCP	7.09 ± 1.6 a	6.8 ± 1.4 a	0.29 ± 0.05b
		with MCP	2.42 ± 0.2 b	2.06 ± 0.1 bc	0.39 ± 0.04 a

## Supplementary Materials

The following are available online at https://www.mdpi.com/2076-2607/7/9/329/s1, Table S1: Physical and chemical soil properties of three different experimental soils.

## References

[1] Bashan Y. (1998). Inoculants of plant growth-promoting bacteria for use in agriculture. Biotechnol. Adv..

[2] Glick B.R. (2014). Bacteria with ACC deaminase can promote plant growth and help to feed the world. Microbiol. Res..

[3] Hartmann A., Schmid M. (2009). Plant-driven selection of microbes. Plant Soil.

[4] Woo S.L., Pepe O., Fertilizers V.S.P. (2018). Microbial Consortia: Promising Probiotics as Plant Biostimulants for Sustainable Agriculture. Front. Plant Sci..

[5] Menzies N., Harbison D., Dart P. Soil chemistry-facts and fiction and their influence on the fertilizer decision making process. Proceedings of the 26th Annual Conference of the Grassland Society of NSW.

[6] Schütz L., Gattinger A., Meier M., Müller A., Boller T., Mäder P., Mathimaran N., Scotti R. (2018). Improving Crop Yield and Nutrient Use Efficiency via Biofertilization - A Global Meta-analysis. Front. Plant Sci..

[7] Rodríguez H., Fraga R. (1999). Phosphate solubilizing bacteria and their role in plant growth promotion. Biotechnol. Adv..

[8] Nuti M., Giovannetti G. (2015). Borderline Products between Bio-fertilizers/Bio-effectors and Plant Protectants: The Role of Microbial Consortia. J. Agric. Sci. Technol. A.

[9] Lopez-Cervantes J., Thorpe D.T. (2013). Microbial Composition Comprising Liquid Fertilizer and Processes for Agricultural Use. U.S. Patent.

[10] Liu H., White P.J., Li C. (2016). Biomass partitioning and rhizosphere responses of maize and faba bean to phosphorus deficiency. Crop Pasture Sci..

[11] Bradáčová K., Kandeler E., Berger N., Ludewig U., Neumann G. (2019). Microbial Consortia Stimulate Early Growth of Maize Depending on N and P Supply. Plant Soil Environm..

[12] VDLUFA (Verband Deutscher Landwirtschaftlicher Untersuchungs-und Forschungsanstalten e.V. Speyer, Germany) (1991). Handbuch der Landwirtschaftlichen Versuchs- und Untersuchungsmethodik Methodenbuch Band I Die Untersuchung von Böden.

[13] Gericke S., Kurmies B. (1952). Die kolorimetrische Phosphorsäure bestimmung mit Ammonium, Vanadat, Molybdat und ihre Anwendung in der Pflanzenanalyse. Z. für Pflanz. Düngung Bodenkd..

[14] Stemmer M. (2004). Multiple-substrate enzyme assays: A useful approach for profiling enzyme activity in soils?. Soil Biol. Biochem..

[15] Chrominski K., Tkacz M. (2010). Comparison of outlier detection methods in biomedical data. J. Med. Inform. Technol..

[16] Campbell R.C. (2009). Reference Sufficiency Ranges for Plant Analysis in the Southern Region of the United States.

[17] Mpanga I.K., Gomez-Genao N.J., Moradtalab N., Wanke D., Chrobaczek V., Ahmed A., Windisch S., Geistlinger J., Walker F., Ludewig U. (2019). The role of N form supply for PGPM-host plant interactions in maize. J. Plant Nutr. Soil Sci..

[18] Park R.M., Hasenstein K.H. (2015). Hormone-Induced Gene Expression During Gravicurvature of Brassica Roots. J. Plant Growth Regul..

[19] Gälweiler L., Guan C., Müller A., Wisman E., Mendgen K., Yephremov A., Palme K. (1999). Regulation of Polar Auxin Transport by AtPIN1 in Arabidopsis Vascular Tissue Regulation of Polar Auxin Transport by AtPIN1 in Arabidopsis Vascular Tissue. Science.

[20] Mpanga I.A., Nkebiwe P.M., Kuhlmann K., Cozzolino V., Piccolo A., Geistlinger G., Berger N., Ludewig U., Neumann G. (2019). The Form of N Supply Determines Plant Growth Promotion by P-Solubilizing Microorganisms in Maize. Microorganisms.

[21] Neumann G., Römheld V., Waisel Y., Eshel A., Kafkafi U. (2002). Root-induced changes in the availability of nutrients in the rhizosphere. Plant Roots the Hidden Half.

[22] Duus J., Lekfeldt S., Rex M., Mercl F., Kulhánek M., Tlustoš P., Magid J. (2016). Effect of bioeffectors and recycled P - fertiliser products on the growth of spring wheat. Chem. Biol. Technol. Agric..

[23] Thonar C., Duus J., Lekfeldt S., Cozzolino V., Kundel D., Kulhánek M., Mosimann C., Neumann G., Piccolo A., Rex M. (2017). Potential of three microbial bio - effectors to promote maize growth and nutrient acquisition from alternative phosphorous fertilizers in contrasting soils. Chem. Biol. Technol. Agric..

[24] Mpanga I.K., Dapaah H.K., Geistlinger J., Ludewig U. (2018). Soil Type-Dependent Interactions of P-Solubilizing Microorganisms with Organic and Inorganic Fertilizers Mediate Plant Growth Promotion in Tomato. Agronomy.

[25] Robinson D., Rorison I.H. (1987). Root hairs and plant growth at low nitrogen availabilities. New Phytol..

[26] Kania A., Guldner M., Szabo B., Kazem S., Römheld V., Neumann G., Morhard J., Evers M., Terlouw T. (2007). Functional characterization of the stabilized organic turf grass fertilizer ‘Marathon’. Rasen. Turf. Gazon..

[27] Patil N.B., Gajbhiye M., Ahiwale S.S., Gunjal A.B., Kapadnis B.P. (2011). Optimization of Indole 3 acetic acid (IAA) production by Acetobacter diazotrophicus L1 isolated from Sugarcane. Int. J. Environ. Sci..

[28] Bharucha U., Patel K., Trivedi U.B. (2013). Optimization of Indole Acetic Acid Production by Pseudomonas putida UB1 and its Effect as Plant Growth-Promoting Rhizobacteria on Mustard (Brassica nigra). Agric. Res..

[29] Ortíz-Castro R., Contreras-Cornejo H.A., Macías-Rodríguez L., López-Bucio J. (2009). The role of microbial signals in plant growth and development. Plant Signal. Behav..

[30] Hartmann A., Rothballer M., Hense B.A., Schröder P. (2014). Bacterial quorum sensing compounds are important modulators of microbe-plant interactions. Front. Plant Sci..

[31] Sharifi R., Ryu C. (2018). Revisiting bacterial volatile-mediated plant growth promotion: Lessons from the past and objectives for the future. Ann. Bot..

[32] Petrášek J., Friml J. (2009). Auxin transport routes in plant development. Development.

[33] Garnica-Vergara A., Barrera-Ortiz S., Mu E., Raya-gonz J. (2015). The volatile 6-pentyl-2H-pyran-2-one from Trichoderma atroviride regulates Arabidopsis thaliana root morphogenesis via auxin signaling and ETHYLENE INSENSITIVE 2 functioning. New Phytol..

[34] Vinci G., Cozzolino V., Mazzei P., Monda H., Spaccini R., Piccolo A. (2018). An alternative to mineral phosphorus fertilizers: The combined effects of Trichoderma harzianum and compost on Zea mays, as revealed by 1 H NMR and GC-MS metabolomics. PLoS ONE.

[35] Vinci G., Cozzolino V., Mazzei P., Monda H., Savy D., Drosos M., Piccolo A. (2018). Effects of Bacillus amyloliquefaciens and different phosphorus sources on Maize plants as revealed by NMR and GC-MS based metabolomics. Plant Soil.

[36] Bradáčová K., Florea A.S., Bar-Tal A., Minz D., Yermiyahu U., Shawahna R., Kraut-Cohen J., Zolti A., Erel R., Dietel K. (2019). Microbial Consortia versus Single-Strain Ionculants: An Advantage in PGPM-Assisted Tomato Production?. Agronomy.

[37] Benckiser G., Christ E., Herbert T., Weiske A., Blome J., Hardt M. (2013). The nitrification inhibitor 3, 4-dimethylpyrazole-phosphat (DMPP)- quantification and effects on soil metabolism. Plant Soil.

[38] Bradáčová K., Weber N.F., Talab N.M., Asim M., Imran M., Weinmann M., Neumann G. (2016). Micronutrients (Zn/Mn), seaweed extracts, and plant growth - promoting bacteria as cold - stress protectants in maize. Chem. Biol. Technol. Agric..

[39] Moradtalab N., Weinmann M., Walker F., Höglinger B., Ludewig U., Neumann G. (2018). Silicon Improves Chilling Tolerance During Early Growth of Maize by Effects on Micronutrient Homeostasis and Hormonal Balances. Front. Plant Sci..

[40] Kochian L.V., Pin M.A., Hoekenga O.A. (2005). The physiology, genetics and molecular biology of plant aluminum resistance and toxicity. Plant Soil.

[41] Britto D.T., Kronzucker H.J. (2002). Review NH4+ toxicity in higher plants: A critical review. J. Plant Physiol..

[42] Emanuelsson J. (1984). Root growth and calcium uptake in relation to calcium concentration. Plant Soil.

[43] Njoku B.O., Enwezor W.O., Onyenakwe B.I. (1987). Calcium deficiency identified as an important factor limiting maize growth in acid ultisols of eastern Nigeria Laboratory incubation. Fertil. Res..

[44] Jakli-Hauer M., Tränkner M. (2019). Critical Leaf Magnesium Thresholds and the Impact of Magnesium on Plant Growth and Photo-Oxidative Defense: A Systematic Review and Meta-Analysis from 70 Years of Research. Front. Plant Sci..

[45] Jing J., Rui Y., Zhang F., Rengel Z., Shen J. (2010). Localized application of phosphorus and ammonium improves growth of maize seedlings by stimulating root proliferation and rhizosphere acidification. Field Crops Res..

[46] Nkebiwe P.M., Weinmann M., Müller T. (2016). Improving fertilizer-depot exploitation and maize growth by inoculation with plant growth-promoting bacteria: From lab to field. Chem. Biol. Technol. Agric..

