# Supplementary material for: Maize Inoculation with Microbial Consortia: Contrasting Effects on Rhizosphere Activities, Nutrient Acquisition and Early Growth in Different Soils
Source: Microorganisms. 2019 Sep 7;7(9):329. doi: 10.3390/microorganisms7090329 (PMC6780557; doi:10.3390/microorganisms7090329)
Supplement: Supplementary file 1 [file microorganisms-07-00329-s001.zip › Supplementary/Figure_S1.docx]

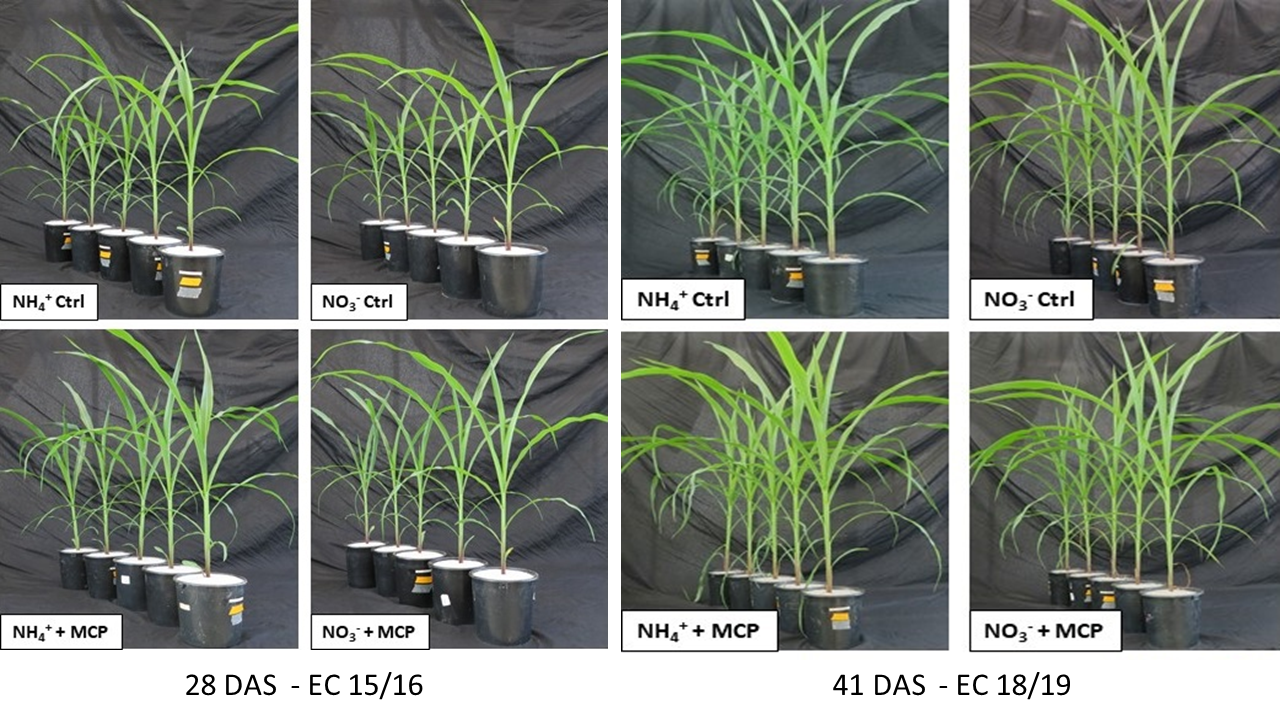


Figure S1: Habitus of maize plants (cv Jessy) after a culture period of 28 days after sowing (DAS) and 41 DAS on a silty-loam field soil (pH 5.9) with low P availability (Soil 2) supplied with moderate soluble P fertilization (30 mg P kg^-1)^ and N in form of Ca-nitrate (NO_3_^-^) or DMPP-stabilized ammonium (NH_4_^+^) with (MCP) and without MCP inoculation (Ctrl). EC = developmental stage.
