# Supplementary material for: Maize Inoculation with Microbial Consortia: Contrasting Effects on Rhizosphere Activities, Nutrient Acquisition and Early Growth in Different Soils
Source: Microorganisms. 2019 Sep 7;7(9):329. doi: 10.3390/microorganisms7090329 (PMC6780557; doi:10.3390/microorganisms7090329)
Supplement: Supplementary file 1 [file microorganisms-07-00329-s001.zip › Supplementary/Table_S1.docx]

**Table S1.** Physical and chemical soil properties of three different experimental soils.

|  | **Soil 1** | **Soil 2** | **Soil 3** |
| --- | --- | --- | --- |
| **Soil Origin** | **Sandy**  **Soil mixture** | **Freshly derived field soil**  **Heßberg, Germany** | **Calcareous Loess sub-soil Wippenhausen, Germany** |
| **Soil properties** | Sandy-loam | Clay-loam | Loam |
| Soil pH (CaCl_2_) | 6.1 | 5.9 | 7.6 |
| Total Nitrogen [%] | 0.076 | 0.26 | 0.03 |
| Plant available P (P CAL) [mg kg^-1^ soil] | 7 | 20 | 5 |
| K (CAL) [mg kg^-1^ soil] | 72.2 | 38.2 | 47.0 |
| Mg [mg kg^-1^ soil] | 230 | 230 | 200 |
| Total carbon C-org [%] | 0.58 | 2.24 | 0.16 |
