# Supplementary material for: Maize Inoculation with Microbial Consortia: Contrasting Effects on Rhizosphere Activities, Nutrient Acquisition and Early Growth in Different Soils
Source: Microorganisms. 2019 Sep 7;7(9):329. doi: 10.3390/microorganisms7090329 (PMC6780557; doi:10.3390/microorganisms7090329)
Supplement: Supplementary file 1 [file microorganisms-07-00329-s001.zip › Supplementary/Table_S2.docx]

**Table S2.** Overview on the experimental setups employed for maize cultivation

|  | Soil 1 | Soil 2 | Soil 3 |
| --- | --- | --- | --- |
| Soil Origin  Soil Type  Soil pH (CaCl_2_)  Soil storage  Substrate [kg pot^-1^]  (soil 70/sand 30% w/w)  Number of plants pot^-1^ (directly sown)  Replicates / treatment  Duration of the experiment  N fertilization  [mg N kg^-1^ substrate]  P fertilization  [mg P kg^-1^ substrate]  K fertilization  [mg K kg^-1^ substrate]  Mg fertilization  [mg Mg kg^-1^ substrate] | Soil mixture  Sandy loam  6.1  >20 years air-dried  2.4  1  5  35 d  Ca(NO_3_)_2_ 140 or  (NH_4_)_2_SO_4_+DMPP 140  Soluble Ca(H_2_PO_4_)_2_ 30  K_2_SO_4_ 150  MgSO_4_ 50 | Freshly derived field soil  Ap-Horizon Heßberg, Germany  Clay Loam  5.9  2 months air-dried  2.9  1  5  28 d; 41 d  Ca(NO_3_)_2_ 140 or  (NH_4_)_2_SO_4_+DMPP 140  Soluble  Ca(H_2_PO_4_)_2_ 30  K_2_SO_4_ 150  MgSO_4_ 50 | Calcareous Loess subsoil,  C-Horizon  Wippenhausen, Germany  Clay  7.6  >20 years air-dried  1.3  1  10  37 d  Ca(NO_3_)_2_ 140 or  (NH_4_)_2_SO_4_+DMPP 140  Insoluble  Rock-Phosphate 80  K_2_SO_4_ 150  MgSO_4_ 50 |
