# Supplementary figures and images for: Maize Inoculation with Microbial Consortia: Contrasting Effects on Rhizosphere Activities, Nutrient Acquisition and Early Growth in Different Soils
Source: Microorganisms. 2019 Sep 7;7(9):329. doi: 10.3390/microorganisms7090329 (PMC6780557; doi:10.3390/microorganisms7090329)

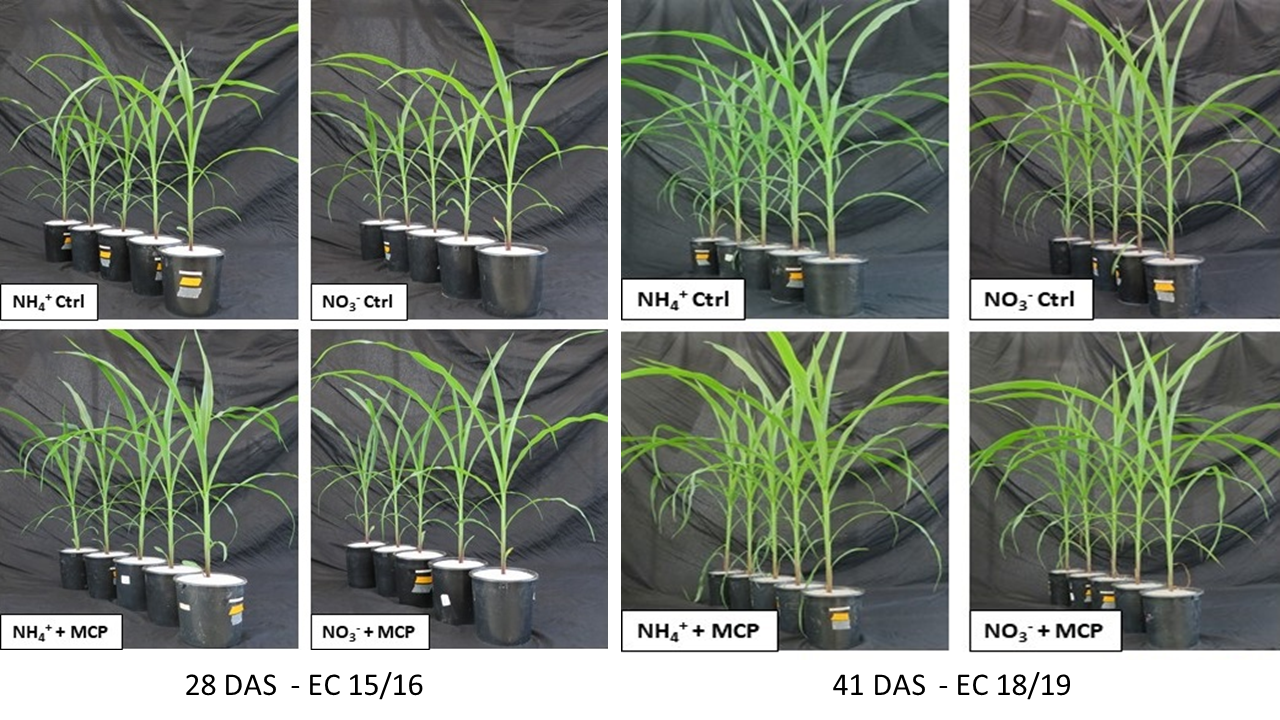

Supplement: Supplementary file 1 [file microorganisms-07-00329-s001.zip › Supplementary/Fig_S1.bmp]
